# Influence of a Virgin Olive Oil versus Butter Plus Cholesterol-Enriched Diet on Testicular Enzymatic Activities in Adult Male Rats

**DOI:** 10.3390/ijms18081701

**Published:** 2017-08-04

**Authors:** Germán Domínguez-Vías, Ana Belén Segarra, Magdalena Martínez-Cañamero, Manuel Ramírez-Sánchez, Isabel Prieto

**Affiliations:** Department of Health Sciences, Unit of Physiology, University of Jaen, 23071 Jaén, Spain; german.dominguez@uca.es (G.D.-V.); asegarra@ujaen.es (A.B.S.); canamero@ujaen.es (M.M.-C.); msanchez@ujaen.es (M.R.-S.)

**Keywords:** testis-peptidase-activity, renin-angiotensin-system, high-fat-diet, virgin-olive-oil

## Abstract

The aim of the present work was to improve our knowledge on the mechanisms underlying the beneficial or deleterious effects on testicular function of the so-called Mediterranean and Western diet by analyzing glutamyl aminopeptidase (GluAP), gamma glutamyl transpeptidase (GGT) and dipeptidyl peptidase IV (DPP IV) activities in testis, as enzymes involved in testicular function. Male Wistar rats (6 months old) were fed for 24 weeks with three different diets: standard (S), an S diet supplemented with virgin-olive-oil (20%) (VOO), or a S diet enriched with butter (20%) plus cholesterol (0.1%) (Bch). At the end of the experimental period, plasma lipid profiled (total triglycerides, total cholesterol and cholesterol fractions (HDL, LDL and VDL)) were measured. Enzymatic activities were determined by fluorimetric methods in soluble (sol) and membrane-bound (mb) fractions of testicular tissue using arylamide derivatives as substrates. Results indicated an increase in plasmatic triglycerides, total cholesterol, LDL and VLDL in Bch. A significant increase of mb GluAP and GGT activities was also found in this diet in comparison with the other two diets. Furthermore, significant and positive correlations were established between these activities and plasma triglycerides and/or total cholesterol. These results support a role for testicular GluAP and GGT activities in the effects of saturated fat (Western diet) on testicular functions. In contrast, VOO increased sol DPP IV activity in comparison with the other two diets, which support a role for this activity in the effects of monounsaturated fat (Mediterranean diet) on testicular function. The present results strongly support the influence of fatty acids and cholesterol on testicular GluAP and GGT activities and also provide support that the reported beneficial influence of the Mediterranean diet in male fertility may be mediated in part by an increase of testicular sol DPP IV activity.

## 1. Introduction

High fat diets have been related to obesity and to several associated health problems such as hypertension, atherosclerosis, diabetes and infertility [1]. Dietary fatty acid composition modifies sex steroid levels and influences fertility in mammals. However, the results are still not conclusive, and the involved mechanisms are not totally understood [2]. It has been reported that hypercholesterolemia and high plasma levels of triglycerides are associated with poor semen quality and direct adverse effects on the testicular function that may lead to male infertility [3]. Hypercholesterolemic rats have a marked decrease in fertility index, testicular weight, sperm cell count, and percentages of sperm motility and viability, associated with a significant increase in sperm cell abnormalities [1]. Diets rich in saturated fatty acids modify the testicular morphology, with lower seminiferous epithelium height, diameter and cell proliferation [4]. In addition, a 20% supplementation of fat in the diet during sexual maturation markedly reduces the daily sperm production at adulthood in Wistar rats [5].

Lipids have been closely associated with intracellular generation of reactive oxygen species [2,6] as substrates for oxidative attack and a source of free radical generation and/or chain propagator reactions. In addition, different oil-supplemented diets strongly modify testicular lipid composition [1,2], and this is closely related to changes in several enzymatic activities that play a major role in the metabolism of bioactive peptides. In addition, it has been observed that the function of the hypothalamic–pituitary–gonadal axis is highly sensitive to adverse metabolic conditions such as obesity and diabetes. Obesity in men is associated with lower total testosterone, free testosterone and sex hormone-binding globulin, and the decline in testosterone and free testosterone with age is greater in obese compared with lean men [7]. On the other hand, it has been reported that cholesterol-enriched diet disrupts the blood testis barrier and increases lipid deposition in the seminiferous tubules [8].

A local renin-angiotensin system (RAS) has been clearly established in testis which may participate in male reproductive functions [9,10,11]. It has been suggested that Ang II-metabolizing activity (glutamyl aminopeptidase, GluAP) may be influenced by dietary fatty acids composition and cholesterol content during early mice development [12]. This assumption was speculative and based on various independent studies [13,14,15] which, taken together, would suggest such a conclusion. In the present study and in order to analyze the influence of those factors, we simultaneously analyzed testicular GluAP, as well as two other recognized enzymes involved in the male reproductive function, such as gamma glutamyl transpeptidase (GGT) and dipeptidyl peptidase IV (DPP IV) [16,17], together with plasma lipid profile: total triglycerides, total cholesterol and cholesterol fractions (HDL, LDL and VLDL), in animals fed with three different diets: (1) standard (S), (2) an S diet supplemented with virgin-olive-oil (20%) (VOO) and (3) an S diet enriched with butter (20%) plus cholesterol (0.1%) (Bch). In addition, in order to compare with a previous study [12] in which the period of feeding was performed during early mice development (from 1–2 weeks-old to 11–12 weeks-old), the present study was carried out at the beginning of the feeding period in fully developed 6-month-old adult male Wistar rats, which were fed for 6 additional months, being sacrificed and obtained their testicular and plasma samples when they reached 12 months of age.

## 2. Results

### 2.1. Body Weight

**Figure 1:**
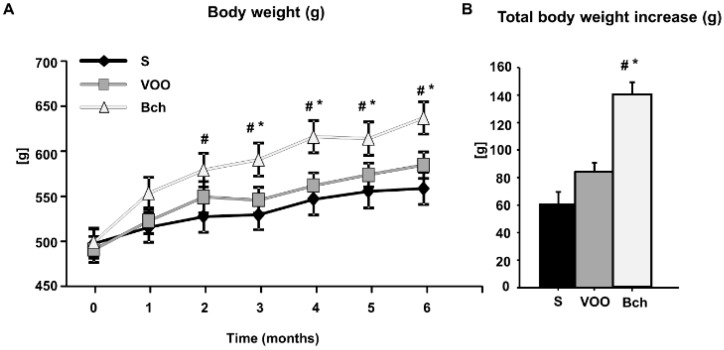
(**A**) Values represent mean ± SEM of body weight (g) in a serial time analysis, during the 6 months of the experimental period: from 0 (6 months-old) to 6 (12 months-old) in animals fed standard (S), virgin olive oil (VOO) and butter plus cholesterol (Bch) diets. (**B**) Mean ± SEM values of body weight increase (g) in S, VOO and Bch. * (*p* < 0.05) differences vs. standard diet, # (*p* < 0.05) differences vs. virgin olive oil diet.

Animals fed the Bch diet showed higher body weight compared with S and VOO groups from the respective 2nd and 3rd month of the feeding period. However, no significant differences were observed between S and VOO diets throughout the experimental period (Figure 1A). At the end of the experimental period, the body weight increase (g/day) was 61.32 ± 8.39 (mean ± SEM) for the S diet and 84.21 ± 6.71 for the VOO diet (37.33% higher than S group) without significant differences between them. However, the mean ± SEM body weight increase for Bch was 140.08 ± 8.92 g/day, which was significantly (*p* < 0.05) different in comparison with the S and VOO groups (128.44% higher than S and 65.56% higher than VOO) (Figure 1B).

### 2.2. Lipid Plasma Profile

**Figure 2:**
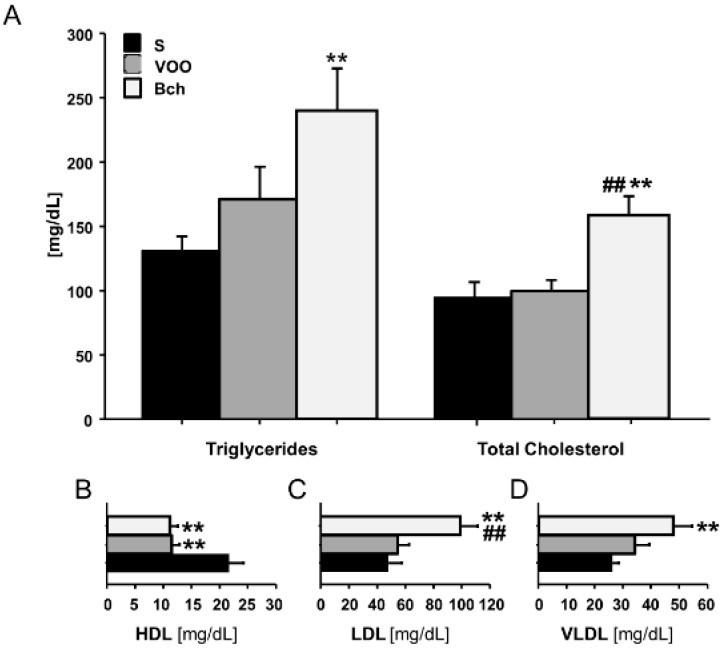
Values represent mean ± SEM levels, expressed in mg/dL, of plasma total triglycerides and cholesterol (**A**), and HDL (**B**), LDL (**C**) and VLDL (**D**) cholesterol fractions in standard (S), virgin olive oil (VOO) and butter plus cholesterol (Bch) diets. ** (*p* < 0.01) differences vs. standard diet, ## (*p* < 0.01) differences vs. virgin olive oil diet.

Figure 2 shows the plasmatic values of triglycerides, total cholesterol, HDL, LDL and VLDL cholesterol in animals fed the S, VOO and Bch diets. Results indicated highly significant differences in triglycerides (*p* < 0.01), total cholesterol (*p* < 0.01), LDL (*p* < 0.01) and VLDL cholesterol (*p* < 0.01). While triglycerides were higher in Bch in comparison with S, without differences compared with VOO, total cholesterol was higher in Bch than in S and VOO (Figure 2A). HDL was significantly higher in S in comparison with VOO and Bch, LDL was higher in Bch in comparison with VOO and S, and VLDL was higher in Bch only in comparison with S (Figure 2B).

### 2.3. GluAP, GGT and DPP-IV Activities

**Figure 3:**
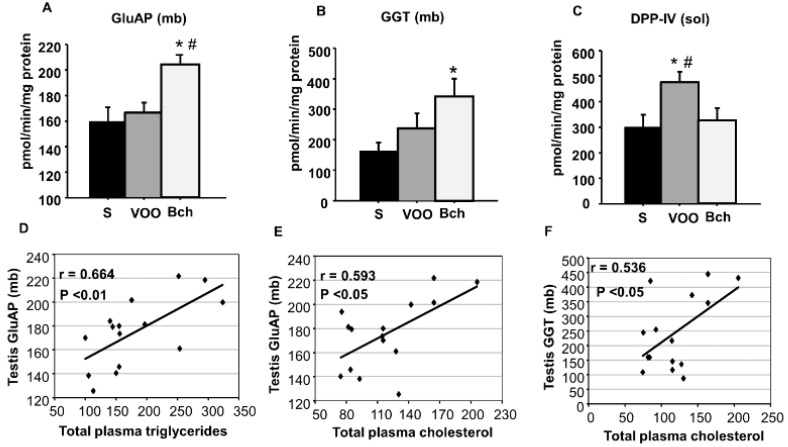
Values represent mean and SEM levels of membrane bound (mb) GluAP activity (**A**), mb GGT activity (**B**) and soluble (sol) DPP-IV activity (**C**) in animals fed standard (S), virgin olive oil (VOO) and butter plus cholesterol (Bch) diets. Values are expressed as pmol of the corresponding aminoacyl-β-naphtylamide hydrolyzed per minute and per mg of protein. * (*p* < 0.05), differences vs. standard diet. # (*p* < 0.05) differences vs. butter plus cholesterol diet. The significant correlations that were observed when testicular sol or mb enzymatic activities and plasmatic lipids were compared are also represented (**D**–**F**), indicating r and *p* values.

While GluAP and GGT activities were higher (*p* < 0.05) in Bch than in VOO and S in the mb fraction, no differences were observed between groups in the sol fraction. In contrast, DPP IV activity was higher (*p* < 0.05) in VOO in comparison with S and Bch (Table 1 and Figure 3A–C).

Furthermore, significant and positive correlations were established between mb GluAP activity and total plasma triglycerides (Figure 3D, *p* < 0.01, *r* = 0.664) and total plasma cholesterol (Figure 3E, *p* < 0.05, *r* = 0.593). In addition, a significant positive correlation was established between testicular mb GGT activity and total plasma cholesterol (Figure 3F, *p* < 0.05, *r* = 0.536).

## 3. Discussion

Currently, there is an increasing interest in the relationship between high fat diet, obesity and male infertility, but the proposed mechanisms to explain such association are not yet totally understood [1,2]. Different studies have demonstrated that the increase of plasmatic triglycerides and cholesterol is related to lower semen quality and infertility [18,19]. However, concomitant oral administration of α-tocopherol (antioxidant) and simvastatin (a mild cholesterol lowering agent) to male hypercholesterolemic rats induced an additional protective effect on the reduced male fertility [1]; oleouropein supplementation, a phenolic compound in virgin olive oil, increase testicular testosterone levels in rats fed a high-protein diet [20], and the administration of olive oil to hipercholesterolemic male rabbits recovered the loss of semen quality and sperm functionality [21]. Previous results from our laboratory have demonstrated a differential effect of several fat sources on plasma GluAP activity and testosterone levels in male Wistar rats, indicating the importance of dietary fat components [22]. In addition, it has been reported that extra virgin olive oil reduced the adverse effects of genetically modified soya been on the reproductive organs of adult male rats [23], and it has been suggested that an appropriate mixture of olive oil and soybean oils may improve the redox homeostasis and steroidogenic status in rat testis [24].

The present results confirm the influence of high fat diets on body weight and plasma lipid profile, increasing the levels of triglycerides and cholesterol. Since no significant differences were found in these parameters between the VOO group and the S one, these effects seem to depend on the fat source. In this way, previous results have demonstrated that the dietary fat source and the changes in plasma lipid levels are related to testosterone synthesis [22] and an impaired testicular function, suggesting that hypercholesterolemia is an independent risk factor for testicular dysfunction [25]. In the present research, we found an increase in body weight and plasma lipids (total triglycerides and cholesterol) of animals fed the Bch diet when compared with the S diet, but no significant differences were found between the S and VOO groups. Furthermore, as observed in the present work, the effect on enzyme activities related to fertility was also associated with the dietary fat: whereas Bch increased significantly GluAP and GGT membrane-bound activities, the diet supplemented with VOO increased soluble DPP-IV activity.

In the male reproductive system, the local RAS function is thought to be relevant for fertility [9,11]. It is well known that testicular RAS has been implicated in inhibiting testosterone production during male steroidogenesis [26]. Interestingly, AngII has been shown to inhibit Leydig cell function and is thus implicated in the local regulation of the testis by pituitary luteinizing hormone [27]. AngII inhibits adenylate cyclase activity in Leydig cell membranes and reduces basal chorionic gonadotropin-stimulated cAMP as well as testosterone production. These data suggest that locally produced AngII could negatively modulate luteinizing hormone stimulation of Leydig cells. Ang II is also involved in the paracrine regulation of the seminiferous tubule function because it induces contraction, growth and rise in intracellular calcium in rat peritubular myoid cells via AT1 receptors. High fat diets inhibit the gene and the protein expression of several RAS components in testis, including angiotensin converting enzyme (ACE), renin and AT1 receptor, and enalapril, an ACE inhibitor, reverts these changes [11].

Glutamyl aminopeptidase activity (Aminopeptidase A, EC 3.4.11.7) selectively hydrolyses acidic amino acid residues from the amino terminus of oligopeptides [28], mainly AngII, the most important bioactive peptide of the RAS, through its stimulation of the AT1 receptor. Deletion of the N-terminal Asp residue of Ang II by GluAP results in Ang III, a less potent vasoconstrictor than Ang II [29]. Therefore, the increase of GluAP in testis of Bch and its positive correlation with triglycerides and cholesterol strongly supports its involvement in the mechanisms underlying the effects of this diet in testicular function. However, it should be taken into account that cholecystokinin, another substrate of GluAP [28,29], can be released during the acrosome reaction and therefore, has relevance in the fertilization process [30].

The butter plus cholesterol diet also increased GGT activity (EC 2.3.2.2), an important membrane-bound enzyme involved in glutathione metabolism [31]. In the seminiferous tubule, this enzyme is localized in Sertoli cells. GTT has been considered a marker of Sertoli cell functions [32] and is stimulated by FSH and germ cell-secreted products [33]. Previous studies have shown that GGT is present in epididymal epithelial cells and luminal fluids [33]. It has been proposed that epididymal GGT may have a role in protecting spermatozoa from oxidative stress in the epididymal duct and/or recover extracellular cysteine for the synthesis of epididymal proteins [33]. Our results demonstrated that a high saturated fat diet is able to modulate testicular GGT activity. The high mb GGT activity under the BCh diet may be a significant sign of greater protection from the elevated oxidative stress provoked by lipid peroxidation.

Dipeptidyl peptidase IV activity may influence testis development/spermatogenesis by regulating the immune state in the testis. In addition, DPP-IV is also known as the T-cell activation antigen CD26 [34]. DPP-IV and DPP-VIII are considered as co-stimulatory of T-cells to participate in immunoreaction [35]. It was reported that DPP-IV probably influences chemotactic activity of some chemokines on Th2 lymphocytes and dendritic cells, but not on Th1 lymphocytes. Furthermore, the immunoregulatory role of these enzymes may be especially important in testes. This is in accordance with the accepted idea that immunoreaction must attain a balance between immune resistance and immune protection in testes, which contributes to the maintenance of normal spermatogenesis. Our results indicated that high degradation of substrate Gly-Pro-peptides with the VOO diet might keep a better balance in the process of spermatogenesis, which implies high activity within the DPP-IV family [17], and it is in agreement with the beneficial effect of antioxidant virgin olive oil components on testosterone production in testis [21].

In order to analyze the influence of different diets on testicular function, the experiments performed by Arechaga et al [12] were carried out only during the development of the reproductive capacity in mice, from 1 and 2 to 11 and 12 weeks of age, equivalent to 9 years of age in humans [36]. In contrast, in order to analyze possible differences, in the present work, the experiments were performed on adult male rats with full reproductive capacity, from 6 months old to 12 months old, equivalent to humans aged 46 years old [37].

The present results, in comparison with a previous report [12], demonstrate that testicular GluAP increased depending on the degree of saturation of the diet but independently of the type of saturated diet and also independently of the fact that the animal was not yet reproductively mature or fully active for reproduction.

In summary, the present results: (1) strongly support the significant relationship between the composition of lipids in the diets, the plasmatic level of lipids in animals fed these diets, the body weight increase and the activity of certain enzymatic activities, (2) demonstrate that, depending on the type of diet, certain enzymatic activities involved in testicular function are selectively activated, which may be useful in order to consider some diets as complementary therapeutic factors to improve some reproductive disorders but also to consider selective activators or inhibitors of these enzymes in therapeutic strategies and (3) indicate that the VOO and Bch diets clearly differ in their possible beneficial or deleterious effects on physiology, including testicular functions.

The present results help to understand the mechanisms by which some type of diets such as VOO or Bch may have beneficial or deleterious influences, respectively, on testicular function.

## 4. Materials and Methods

### 4.1. Animals

Fifteen 6-month-old male Wistar rats, supplied by Harlan Ibérica (Barcelona, Spain), were used in this study. Mean body weight was 495 g at the start of the study. Rats were housed at a constant temperature (25 °C) and under constant day length (12 h). Experimental procedures for animal use and care were in accordance with European Communities Council Directive 2010/63/UE and Spanish regulation RD 53/2013, and the study was approval by the Institutional Animal Care and Use Committee of the University of Jaén.

### 4.2. Experimental Design and Diets

After reception, rats (aged 6 months) were allowed free access to water and food for 24 weeks until the end of the experiment (aged 12 months). Until reception, all rats had been fed with the same standard diet that we used for group 1. Rats were randomly divided into 3 groups (*n* = 5 each). Group 1 (S) was fed a standard diet for experimental rats. Group 2 (VOO) was fed a diet supplemented with virgin olive oil (20%), and group 3 (BCh) was fed a diet supplemented with butter (20%) plus cholesterol (0.1%). The nutrition value and lipid profile of diets used during the experimental periods are shown in Table 2. The standard (S) diet was low in energy and high in carbohydrates compared with the VOO and Bch diets. The virgin olive oil (VOO) diet was high in monounsaturated fatty acids (MUFA) and polyphenols, and the butter plus cholesterol (Bch) diet was high in saturated fatty acids (SFA) and cholesterol.

### 4.3. Samples

After the feeding period (24 weeks), animals were perfused with saline solution through the left cardiac ventricle under equithensin anesthesia (2 mL/kg body weight); a sample of blood was previously removed and centrifuged for 10 min at 2000 g to obtain the plasma.

Samples from the right testis (interstitial area and seminiferous tubules) were quickly removed and frozen in dry ice. To obtain the soluble fraction, tissue samples were homogenized in 10 volumes of 10 mM HCl-Tris buffer (pH = 7.4) and ultracentrifugated at 100,000× *g* for 30 min (4 °C). The resulting supernatants were used to measure soluble enzymatic activity and protein content, assayed in triplicate. To solubilize membrane proteins, pellets were re-homogenized in HCl-Tris buffer (pH = 7.4) plus 1% Triton X-100. After centrifugation (100,000× *g*, 30 min, 4 °C), supernatants were used to measure membrane-bound activity and proteins, also in triplicate. To ensure the complete recovery of activity, detergent was removed from the medium by adding adsorbent polymeric Biobeads SM-2 (100 mg/mL) (Bio-Rad, Richmond, VA, USA) to the samples, which were shaken for 2 h at 4 °C.

### 4.4. Biochemical Assessment

#### 4.4.1. Triglycerides and Cholesterol Assay

Plasma total cholesterol and triglycerides were determined colorimetrically using a commercial kit (Sigma, St. Louis, MO, USA, 352-50). HDL cholesterol was precipitated with phosphotungstic acid and quantified using the same method. LDL and VLDL cholesterol fractions were calculated using Friedewald’s formula.

#### 4.4.2. Enzymatic Assays

Enzymatic activities were assayed fluorimetrically using aminoacyl-beta naphthylamides (aaNNap) as substrates. GluAP activity was determined using GluNNap as a substrate according to the method of Ramírez et al. [38]. Briefly, 10 μL of each supernatant were incubated for 30 min at 37 °C with 100 μL of substrate solution (100 μM GluNNap, 1.5 mM BSA, 0.65 mM DTT and 50 mM CaCl2 in 50 mM HCl-Tris buffer pH 7.4). DPP-IV activity was determined with H-Gly-l-Pro-1-hydroxy-4-NNA hydrochloride, also called Gly-Pro-NNap, as the substrate: 10 μL of each supernatant was incubated for 30 min at 37 °C with 100 μL of substrate solution (100 μM Gly-Pro-NNap, 1.5 mM BSA and 0.65 mM DTT in 50 mM phosphate buffer, pH 8.3). GGT was determined with γ-GluNNap as the substrate: 10 μL of each supernatant was incubated for 30 min at 37 °C with 100 μL of substrate solution (100 μM γ-GluNNap, 1.5 mM BSA and 0.65 mM DTT in 50 mM phosphate buffer, pH 7.4).

All reactions were stopped by adding 100 μL of 0.1 M acetate buffer (pH 4.2). The amount of β-naphthylamine released as a result of enzymatic activity was measured fluorometrically at 412 nm emission wavelength with 345 nm excitation wavelength.

Specific peptidase activities were expressed as pmol of β-naphthylamine hydrolyzed per minute and per milligram of protein. Fluorogenic assays were linear with respect to time of hydrolysis and protein concentration. Protein concentration was determined according to the method of Bradford [39] with BSA as a standard. All chemical products were supplied by Sigma (St. Louis, MO, USA).

#### 4.4.3. Statistical Analysis

For statistical analysis, we used one-way analysis of variance (ANOVA) with post-hoc comparisons using the LSD test. Differences with *p*-values < 0.05 were considered significant. The Pearson correlation coefficient was used to establish the relationship between peptidase activities and plasma lipid levels. All results were expressed as the mean ± standard error.

## 5. Conclusions

The increase in GluAP activity with Bch and the positive correlation observed between testicular GluAP and plasma triglycerides and cholesterol strongly support its role in testicular functions. Moreover, the increase in GGT activity in the Bch group could be related to higher levels of oxidative stress in the testes of these animals. Interestingly, these results were not found with VOO. The VOO diet, rich in monounsaturated fatty acids and polyphenols, increased the levels of DPP-IV activity related to a better immuno-protection in testis. Taken together, these results could help to explain the adverse consequences of high-saturated fatty acids and cholesterol diets on testicular function, support the benefits of a Mediterranean diet and virgin olive oil, and provide new dietary strategies to improve male fertility.

## Figures and Tables

**Table 1:** Values represent mean ± SEM levels of enzymatic activities, in soluble (sol) and membrane-bound (mb) fractions. Values are expressed as pmol of the corresponding aminoacyl-β-naphylamide hydrolyzed per minute and per mg of protein in the sample. GluAP: glutamyl aminopeptidase; GGT: gamma-glutamyl transpeptidase; DPP-IV: dipeptidyl peptidase IV; S: standard chow diet; VOO: 20% virgin olive oil diet; Bch: 20% butter plus 1% cholesterol diet.

Activity	Fraction	S	VOD	Bch	*p*
GluAP	sol	81.31 ± 10.396	117.88 ± 12.987	90.35 ± 11.804	0.108
	mb	159.89 ± 10.956	166.61 ± 7.936	204.18 ± 7.496	0.011
GGT	sol	45.25 ± 7.671	60.52 ± 8.196	49.14 ± 6.447	0.361
	mb	163.98 ± 27.150	236.83 ± 50.370	341.62 ± 59.247	0.046
DPP-IV	sol	302.29 ± 47.355	476.63 ± 41.176	326.55 ± 48.995	0.043
	mb	425.90 ± 56.707	453.29 ± 87.556	593.78 ± 90.857	0.297

**Table 2:** Nutritional value and lipid profile of diets used during the study: S (standard chow diet), VOO (20% virgin olive oil), Bch (20% butter plus 1% cholesterol). Values are expressed as g/100 g of food KJ/100 g of diet and % total energy intake. NFE: Nitrogen Free Extract; SFA: Saturated Fatty Acids; MUFA: Monounsaturated Fatty Acids; PUFA: Polyunsaturated Fatty Acids.

Components	S			VOO			Bch		
	g/100 g	KJ/100 g	Total E %	g/100 g	KJ/100 g	Total E %	g/100 g	KJ/100 g	Total E %
NFE	60	1003.2	72.1	48	802.6	43.4	48	802.6	43.9
Fiber	4			3			3		
Protein	16.5	275.9	19.8	13	217.4	11.8	14	234.1	12.8
Minerals (Ash)	5			5			5		
Lipids	3	112.9	8.1	22	827.6	44.8	21	790	43.3
SFA	0.75	28.22	2.0	3.74	140.7	7.6	13.02	489.8	26.8
MUFA	0.50	18.81	1.4	17.38	653.8	35.4	7.56	284.4	15.5
PUFA	1.75	65.84	4.7	0.88	33.1	1.8	0.42	15.8	0.9
Cholesterol							1.50		
Polyphenols				0.105					
α-tocopherol				0.050			0.0004		
Moisture	11			9			8		
Total E		1392			1848			1827	

## Abbreviations

DPP-IV	Dipeptidyl Peptidase IV
GGT	Gamma Glutamyl Transpeptidase
HDL	High Density Lipoprotein
LDL	Low Density Lipoprotein
VDL	Very Low Density Lipoprotein
BTB	Blood Testis Barrier
RAS	Renin Angiotensin System
AngII	Angiotensin II
Bch	Butter plus cholesterol Diet
S	Standard Diet
VOO	Virgin Olive Oil Diet
GluAP	Glutamyl Aminopeptidase
Sol	Soluble fraction
Mb	Membrane Bound fraction
AngIII	Angiotensin III
NFE	Nitrogen Free Extract
SFA	Saturated Fatty Acids
MUFA	Monounsaturated Fatty Acids
PUFA	Polyunsaturated Fatty Acids
GluNNap	Glutamyl-β-naphthylamine
Gly-Pro-NNap	Glycine-Proline-β-naphthylamine
γ-GluNNap	γ-Glutamyl-β-naphthylamine
BSA	Bovine Serum Albumin
DTT	Dithiothreitol
